# Premyelinating Oligodendrocytes: Mechanisms Underlying Cell Survival and Integration

**DOI:** 10.3389/fcell.2021.714169

**Published:** 2021-07-21

**Authors:** Ethan G. Hughes, Michael E. Stockton

**Affiliations:** Department of Cell and Developmental Biology, School of Medicine, University of Colorado, Aurora, CO, United States

**Keywords:** oligodendrial differentiation, premyelinating oligodendrocytes, myelin, multiple sclerosis, oligodendrocyte precursor cell, oligodendrocyte, survival, integration

## Abstract

In the central nervous system, oligodendrocytes produce myelin sheaths that enwrap neuronal axons to provide trophic support and increase conduction velocity. New oligodendrocytes are produced throughout life through a process referred to as oligodendrogenesis. Oligodendrogenesis consists of three canonical stages: the oligodendrocyte precursor cell (OPC), the premyelinating oligodendrocyte (preOL), and the mature oligodendrocyte (OL). However, the generation of oligodendrocytes is inherently an inefficient process. Following precursor differentiation, a majority of premyelinating oligodendrocytes are lost, likely due to apoptosis. If premyelinating oligodendrocytes progress through this survival checkpoint, they generate new myelinating oligodendrocytes in a process we have termed integration. In this review, we will explore the intrinsic and extrinsic signaling pathways that influence preOL survival and integration by examining the intrinsic apoptotic pathways, metabolic demands, and the interactions between neurons, astrocytes, microglia, and premyelinating oligodendrocytes. Additionally, we will discuss similarities between the maturation of newly generated neurons and premyelinating oligodendrocytes. Finally, we will consider how increasing survival and integration of preOLs has the potential to increase remyelination in multiple sclerosis. Deepening our understanding of premyelinating oligodendrocyte biology may open the door for new treatments for demyelinating disease and will help paint a clearer picture of how new oligodendrocytes are produced throughout life to facilitate brain function.

## Introduction

Oligodendrocytes, the myelin forming cells of the central nervous system, increase the propagation speed of axon potentials and provide support to neurons through ensheathing axons with myelin. While the generation of myelin primarily occurs during development, many regions of the central nervous system continue to undergo active myelination well into adulthood. For example, some axonal tracts, such as the motor root fibers of the spinal cord, are completely myelinated before birth, while other regions such as the neocortex are not fully myelinated until well into the third decade of life in humans (Flechsig, 1901; Yakovlev and Lecours, 1967). Once formed, oligodendrocytes have a remarkable lifespan, surviving for decades in humans (Yeung et al., 2014) and months to years in mice (Tripathi et al., 2017).

**FIGURE 1:**
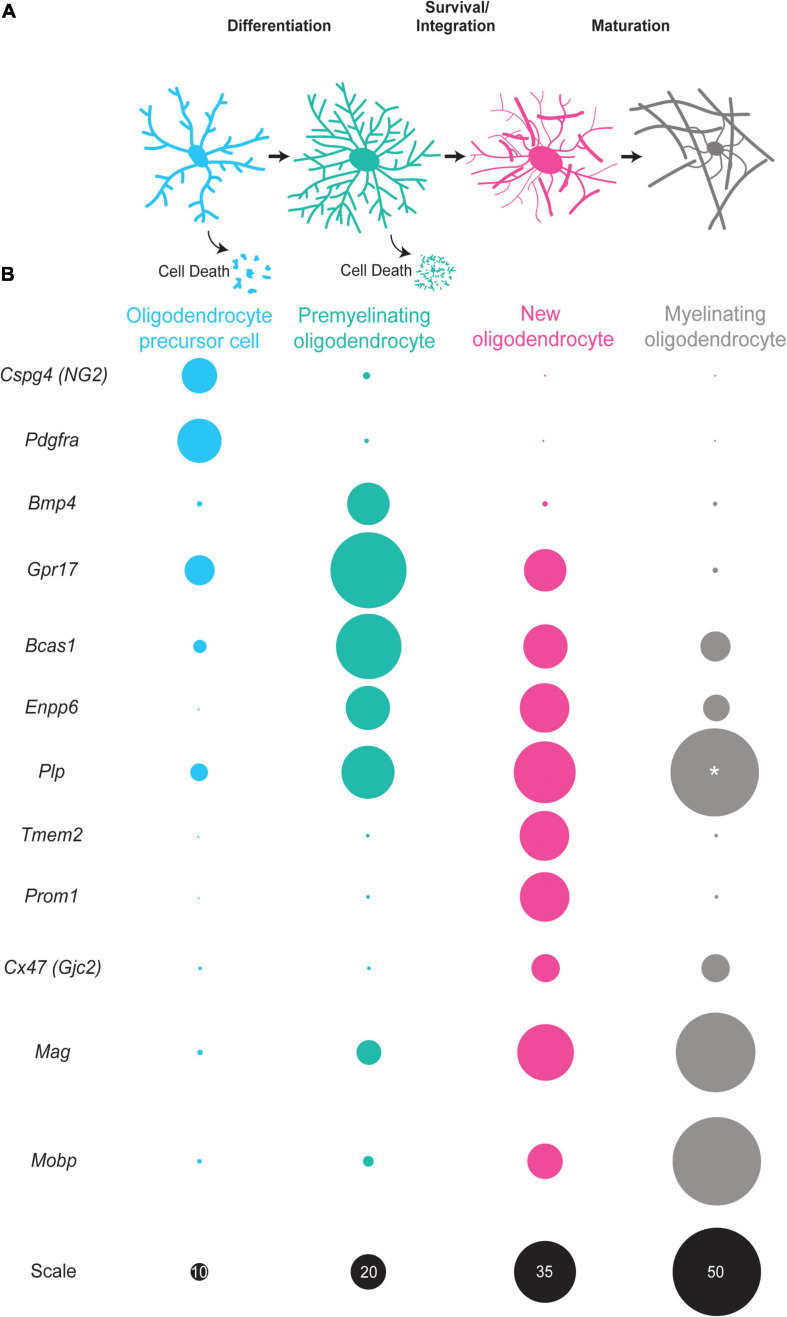
The oligodendrocyte lineage progression and expression of stage-specific markers. **(A)** Oligodendrocyte precursor cells (blue) differentiate into premyelinating oligodendrocytes (teal). A large majority of premyelinating oligodendrocytes are lost but some survive and integrate into neuronal circuits as new oligodendrocytes (pink). New oligodendrocytes then have the capacity to form myelin sheaths and slowly mature into myelinating oligodendrocytes (gray). **(B)** Oligodendrocyte precursor cells (blue) express *Cspg4* and *Pdgfra* mRNA in high levels. Premyelinating oligodendrocytes (teal) have increased expression of *Bmp4*, *Gpr17, Bcas1*, and *Enpp6*. New oligodendrocytes (pink) express some of the same mRNAs as premyelinating oligodendrocytes, yet are distinct as they express *Tmem2*, *Prom2*, and *Cx47* and have increased expression of myelin genes, such as *Plp*, *Mobp*, and *Mag*. Myelinating oligodendrocytes (gray) have high expression of myelin genes, such as *Plp*, *Mobp*, and *Mag* with little to no expression of *Cspg4*, *Gpr17*, and *Enpp6* among others. Size of circles are relative to one another except for those labeled with “*” scale is given in black on the bottom row. The sizes of circles correlate with single cell RNA -sequencing data (Marques et al., 2016).

While most regions of the brain and spinal cord are fully myelinated by the third decade of life in humans, evidence indicates that oligodendrocyte number continues to slowly increase with age (Yakovlev and Lecours, 1967; Peters and Sethares, 2004). Indeed, recent studies utilizing heavy carbon dating from nuclear bomb tests show that some brain regions, such as the adult cerebral cortex, continue to generate new oligodendrocytes throughout life (Yeung et al., 2014). The formation of new myelin-forming oligodendrocytes is called oligodendrogenesis, which is a stepwise differentiation process consisting of three canonical stages: the oligodendrocyte precursor cell (OPC), the premyelinating oligodendrocyte (preOL), and the mature, myelinating oligodendrocyte (OL) (Figure 1). While there has been considerable work studying the cell biology of OPC functions (Bergles and Richardson, 2016; Thornton and Hughes, 2020) and myelination (Stadelmann et al., 2019), considerably less is known about premyelinating oligodendrocyte biology.

Premyelinating oligodendrocytes constitute a population of terminally differentiated cells that are not OPCs but have not yet started to form myelin sheaths. Premyelinating oligodendrocytes, as they are currently defined, were first described in the late 1980s and early 1990s. Immunocytochemical analysis identified a population of highly branched immature cells that were present during peak oligodendrogenesis and myelination (LeVine and Goldman, 1988). These observations prompted the proposal of three stages of the oligodendrocyte differentiation, where the middle stage consists of a morphologically complex, premyelinating cell (Warrington and Pfeiffer, 1992). In the late 1990s, antibodies against DM-20/proteolipid protein (PLP) visualized premyelinating oligodendrocytes contacting unmyelinated axons during developmental myelination (Trapp et al., 1997). Despite the identification and description of premyelinating oligodendrocytes over the last several decades, this stage of oligodendrogenesis has remained relatively poorly defined when compared to OPCs and OLs.

This review will explore how advances in the field of neurogenesis can provide context for the understanding of oligodendrogenesis and premyelinating oligodendrocytes. We will outline what is known about intrinsic and extrinsic control of preOL survival and integration, and discuss how modulation of preOLs has the potential to increase the effectiveness of remyelination therapies in MS.

## Cellular Differentiation: a Perspective From Neurogenesis

Oligodendrogenesis and neurogenesis both produce adult-born, fully functional cells in the mature CNS. However, these processes are both inherently inefficient (Sun et al., 2004; Hughes et al., 2018), with many cells undergoing apoptosis following differentiation (Kawai et al., 2009; Ryu et al., 2016). These similarities, as well as others, suggest that distinct stages of neurogenesis and oligodendrogenesis may be governed by similar processes. Specifically, we will focus on the critical periods of neurogenesis immediately after terminal differentiation of neural progenitors into postmitotic immature neurons. During these critical periods, distinct mechanisms control neuronal survival, integration, and maturation. We will highlight two of these critical periods and discuss how they may also govern the integration and maturation of postmitotic premyelinating oligodendrocytes and new immature oligodendrocytes.

Before discussing critical periods of postmitotic new neuron maturation, it is important to highlight that studies of neurogenesis have been aided in great part by a suite of stage specific markers. These markers have allowed for the identification, and investigation of mechanisms governing cell behavior at distinct stages of the neurogenesis process. However, the identification of proteins and the development of tools to label specific stages of neurogenesis was a gradual process that occurred over many years. Histological studies of mouse and human brain tissue led to the identification of now canonical markers such as DCX, Pax-6, Nestin, GFAP, PSA-NCAM, NeuroD, and Tuj-1 (Lendahl et al., 1990; Doetsch et al., 1997; des Portes et al., 1998; Miyata et al., 1999; Brown J. P. et al., 2003; Encinas and Enikolopov, 2008). During adult neurogenesis, expression of these proteins was found to correlate with distinct stages during proliferation, differentiation, maturation and integration of new neurons (von Bohlen Und Halbach, 2007; Encinas and Enikolopov, 2008; Zhang and Jiao, 2015). Importantly, these stage-specific markers have allowed for detailed studies investigating how disease, life experience, and the local environment influence distinct stages of neurogenesis by different mechanisms. Through the use of stage-specific markers and the discovery of temporally precise critical periods, the integration of adult born neurons in the mature CNS has been thoroughly characterized.

The first critical period in the maturation of new, post-mitotic neurons is a survival checkpoint 1–4 days after differentiation. Here, the apoptotic regulator BAX eliminates a majority of new-born neurons in the sub granular zone of the hippocampus, and genetically eliminating *Bax* expression effectively overrides programmed cell death pathways and increases the number of postmitotic new neurons in the hippocampus (Sun et al., 2004). Furthermore, during normal neurogenesis a majority of newborn cells that have undergone apoptosis are subsequently phagocytosed by microglia (Sierra et al., 2010). Newly generated neurons that pass the survival checkpoint continue to mature and 1–2 weeks after differentiation the second critical period begins.

The second critical period is characterized by the maturation of immature neurons into functional mature cells (Jahn and Bergami, 2018). During this period, postmitotic immature neurons undergo dramatic morphological changes and begin to form and receive synapses (van Praag et al., 1999; Brown J. et al., 2003; Fabel et al., 2009). Furthermore, extrinsic signaling such as exercise (running), exposure to enriched environments, and high-frequency stimulation of the perforant path increase the activation and survival of these neurons (Jungenitz et al., 2014; Jahn and Bergami, 2018). Similar critical periods govern the survival, integration, and maturation of new neurons in other neurogenic niches as well. For example, in the olfactory bulb, sensory input shapes the survival of new granule cells 14–28 days after terminal differentiation (Yamagucho and Mori, 2005), and new olfactory bulb neurons 15–45 days after differentiation (Petreanu and Alvarez-Buylla, 2002). Taken together, these studies and others demonstrate that new-born neurons must override programmed cell death pathways in the first critical period of maturation. During the second critical period, extrinsic factors begin to influence survival and integration over the course of weeks.

Whether critical periods govern the survival, integration, and maturation of oligodendrocytes remains an open question. Recent studies indicate that oligodendrocyte maturation has a similar first temporal critical period, during which many cells are lost shortly after differentiation (Hughes et al., 2018). Furthermore, RNA-sequencing studies have identified multiple populations of newly formed, yet immature, oligodendrocytes in the mouse cortex (Zhang et al., 2014; Marques et al., 2016) suggesting that a second temporal critical period of maturation is shared between oligodendrocytes and neurons. These populations may represent unique stages of oligodendrocyte maturation that are responsive to different extrinsic signals, akin to the maturation of postmitotic new neurons. However, while unique populations of preOLs and new oligodendrocytes have been identified based on RNA expression, the mechanisms governing their integration, survival and maturation have not been as deeply investigated due to a current limited set of reliable tools for visualization and manipulation.

There are a plethora of reliable markers for OPCs, mature oligodendrocytes, and the oligodendrocyte lineage as a whole (Barateiro and Fernandes, 2014). Some examples include PDGFRα, NG2 (also known as Cspg4), MBP, PLP, MAG, MOG, CNPase, OLIG2, and SOX10 (Quarles et al., 1973; Boggs and Moscarello, 1978; Linnington et al., 1984; Vogel and Thompson, 1988; Nishiyama et al., 1996; Kuhlbrodt et al., 1998; Zhou et al., 2000). These markers have been invaluable for studying the biology of OPCs, mature oligodendrocytes and myelin, and have been foundational in characterizing the three canonical stages of oligodendrocyte development. While oligodendrocyte lineage cells have typically been categorized into these three stages as OPCs, preOLs, or mature myelinating oligodendrocytes, it is likely that oligodendrocyte maturation is a developmental continuum rather than distinct stages. Two recent advancements are the identification of breast carcinoma amplified sequence 1 (BCAS1) (Fard et al., 2017) and Ectonucleotide Pyrophosphatase/Phosphodiesterase 6 (ENPP6) as a markers of preOLs and newly integrated oligodendrocytes (Xiao et al., 2016; Figure 1). Once a preOL integrates as a new oligodendrocyte, it experiences dramatic changes in gene expression and function (Figure 1). These new oligodendrocytes continue to add myelin sheaths and eventually become mature, myelinating oligodendrocytes. Interestingly, recent studies show that mature myelinating oligodendrocytes are a heterogenous population of cells with different transcriptional profiles (Marques et al., 2016; Jakel et al., 2019). It remains unclear when and how this heterogeneity arises as the oligodendrocyte precursor populations are transcriptionally homogenous (Marques et al., 2018). It is possible that mature oligodendrocyte heterogeneity arises due to differences in cellular environments or transcriptional heterogeneity within the premyelinating oligodendrocyte stage of the differentiation process. Alternatively, heterogeneity may represent an extended maturation process of mature myelinating oligodendrocytes. The successful use of stage-specific tools in the field of neurogenesis suggests that these approaches will aid in increasing our understanding of the unique stages on the developmental continuum of oligodendrocyte maturation.

## A Bottleneck in the Generation of New Oligodendrocytes

Cell death is required for the normal development of almost all multicellular organisms. During development of the nervous system, apoptosis is necessary to ensure the proper number and location of cells (Haanen and Vermes, 1996). However, whether cell death regulates developmental oligodendrogenesis remained unclear until Barres and colleagues observed that ∼50% of OPCs isolated from the postnatal optic nerve undergo programmed cell death *in vitro* within 2–3 days (Barres et al., 1992). A similar pattern was observed in the cortex, where the percentage of preOLs undergoing degeneration was ∼20% between P7 and P21 and even higher, 37%, at P28 (Trapp et al., 1997). Together, these studies suggest that the developmental programming of oligodendrocytes to undergo apoptosis is an important process in oligodendrocyte development. Oligodendrogenesis is a lifelong process with new oligodendrocytes forming well into adulthood outside normal developmental windows. Are adult born oligodendrocytes also regulated by intrinsic apoptotic signaling? To address this question, Hughes et al. (2018) used *in vivo* two photon imaging to longitudinally track oligodendrocytes in the middle-aged mouse cortex and observed that ∼78% of differentiating OPCs are lost and fail to mature into myelin forming oligodendrocytes. These data demonstrate that the loss of differentiating oligodendrocyte precursors is not confined to typical time course of development; rather, it continues into adulthood and greatly reduces the number of oligodendrocytes available to integrate into the CNS.

The discovery that most oligodendrocytes are lost during the premyelinating oligodendrocyte stage serves to underscore the importance of preOLs in the oligodendrogenesis process. While it is possible that this elimination of preOLs serves some important biological function in adulthood, like ensuring proper levels of myelination, controlling oligodendrocyte number, or removing damaged cells, an alternative explanation is that the loss of preOLs is a continuation of the developmental cell program. Decreasing the number of preOLs eliminated by apoptosis could be beneficial to the adult nervous system to allow for enhanced experience dependent myelination or by providing additional trophic support to axons through the formation of new myelin sheaths. To begin to explore the regulation of this interesting biological process, we will examine the intrinsic and extrinsic factors influencing the survival and integration of oligodendrocytes following precursor differentiation.

## Intrinsic Mechanisms Regulating Premyelinating Oligodendrocyte Survival and Integration

In the adult CNS, the production of new oligodendrocytes is required for learning, memory, and cognition (Xiao et al., 2016; Pan et al., 2020; Wang et al., 2020). However, the profound loss of preOLs during oligodendrogenesis in the adult CNS may limit the rate at which new oligodendrocytes can be produced. Therefore, understanding the intrinsic mechanisms influencing preOLs survival and integration may provide insights into how oligodendrogenesis regulates learning, memory, and cognition.

### Apoptosis and Survival of Premyelinating Oligodendrocytes

After terminal differentiation, oligodendrocyte precursors are postmitotic and enter the preOL stage where they remain for roughly 2 days (Hughes et al., 2018). During this short window of time, many preOLs undergo programmed cell death. Many factors can push cells toward apoptosis including endoplasmic reticulum (ER) and mitochondrial stress. These types of stress are known to induce cell death in a wide range of cell types by regulating Bcl-2 family proteins like BAX and BAK (Zong et al., 2003; Ruiz-Vela et al., 2005). Similar to neurogenesis, BAX and BAK strongly influence apoptosis of differentiating oligodendrocytes. Kawai and colleagues showed that *Bax* and *Bak* mRNAs are expressed at high levels throughout differentiation and maturation of oligodendrocytes *in vitro*. By culturing O4 + oligodendrocytes from mice, they demonstrated that *Bax*–/–*Bak*–/– cultures were resistant to apoptosis after differentiation, with ∼90% of oligodendrocytes remaining viable after 14 days in culture. Cultures from wildtype (WT) mice exhibited apoptosis in almost 90% of cells after just 2 days (Kawai et al., 2009). The significant difference in viability of WT and *Bax*–/–*Bak*–/– cultures illustrates the importance of BAX/BAK signaling in driving apoptosis of differentiating oligodendrocytes. Removal of the BAX/BAK apoptosis checkpoint also resulted in an increased number of mature oligodendrocytes and axons in the optic nerve *in vivo*.

**FIGURE 2:**
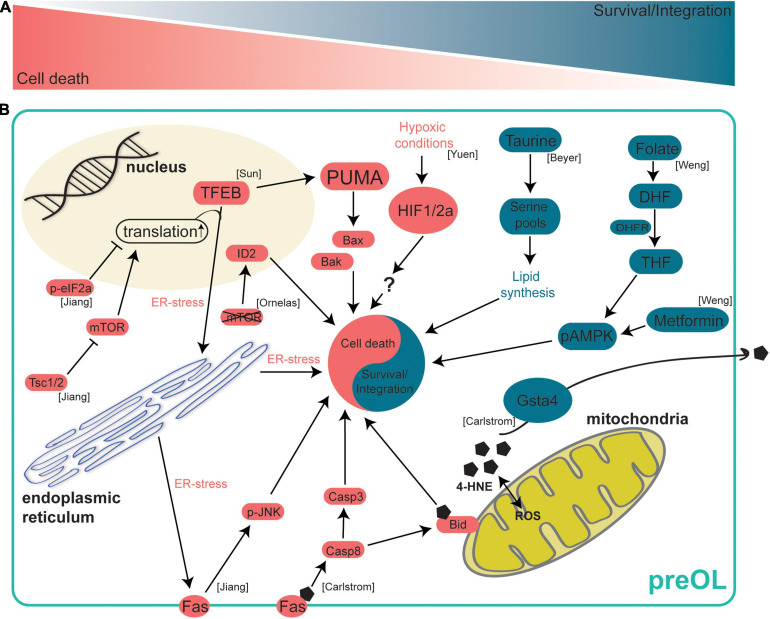
Intrinsic mechanisms regulating preOL survival and integration. After terminal differentiation from OPCs, preOLs must pass through a survival checkpoint during which a majority are lost. At this survival checkpoint a complex web of intracellular signaling mechanisms work to either drive preOL death or to encourage survival and integration. **(A)** A schematic representation of the survival checkpoint. PreOLs can either be driven toward cell death (red) or survival and integration (blue). **(B)** Intrinsic signaling pathways in preOLs. Pathways in blue promote the survival and integration of preOLs. The metabolite Taurine leads to increased serine pools that are available for lipid synthesis pathways (Beyer et al., 2018). The metabolite folate promotes survival by activating AMPKα phosphorylation (Weng et al., 2017). Gsta4 regulates oxidative stress via extracellular transport of 4-HNE (Carlstrom et al., 2020). Pathways in red promote cell death in preOLs. Increased 4-HNE load leads to increased Fas-Casp8-Bid signaling (Carlstrom et al., 2020). Hypoxic conditions result in increased HIF1/2a (Yuen et al., 2014). mTOR deletion leads to increased expression of the oligodendrocyte maturation inhibiting promotor ID2 (Ornelas et al., 2020). Reduction of Tsc1/2 results in hyperactive mTOR, increased translation and ER stress, and increased Fas-JNK signaling (Jiang et al., 2016). TFEB promotes ER stress and increases the expression of PUMA which subsequently leads to increased Bax/Bak activity (Sun et al., 2018).

More recently, altering oligodendrocyte apoptosis was found to control oligodendrocyte number in the white and gray matter. Sun and colleagues identified a novel role for Transcription Factor EB (TFEB) in the BAX/BAK mediated cell death pathway in oligodendrocytes (Sun et al., 2018). Using *Tfeb−/−;Olig2-Cre* mice they demonstrated that deletion of *Tfeb* in the oligodendrocyte lineage (TFEB-cKO) resulted in ectopic myelination of the cerebellar molecular layer and increased oligodendrocyte number throughout the brain, including the gray matter. Interestingly, they observed that the percentage of unmyelinated axons in the white matter was unchanged in TFEB-cKO mice suggesting that different mechanisms may control the myelination of nerve fibers in the white and gray matter. To determine how TFEB is increasing oligodendrocyte number they cultured TFEB-cKO oligodendrocytes and live imaged cells, observing that TFEB-cKO oligodendrocytes experience significantly less cell death during differentiation. Using RNA-sequencing, they found that TFEB promotes expression of genes involved in ER stress pathways. Furthermore, they showed that TFEB induces expression of the Bcl-2 family member, Bcl-2-Binding Component 3 (PUMA) which functions through the Bax/Bak signaling pathway to promote apoptosis in oligodendrocytes (Figure 2).

These two studies demonstrate that altering BAX/BAK signaling in preOLs can alter apoptosis both *in vitro* and *in vivo.* While these studies show that reducing preOL apoptosis can increase oligodendrocyte number, it is unclear why there is ectopic myelination in the cortex and cerebellum while the corpus callosum retains normal numbers of myelinated axons. These differences in the patterns of myelination between white and gray matter following modulation of the efficiency of oligodendrogenesis suggest that disparate mechanisms may control the survival and integration of preOLs in different microenvironments. Furthermore, differences between neurons in white and gray matter may be affecting the placement and stabilization of nascent myelin sheaths, which could result in different patterns of intermittent myelination. These are interesting areas of study and future work will provide insight into the mechanistic basis of myelination differences in white and gray matter.

In addition to BAX/BAK signaling, intrinsic apoptotic pathways in preOLs can be modulated by gene expression changes, ER stress, and other proteins such as mammalian target of rapamycin (mTOR). Genetic deletion of mTOR specifically in oligodendrocytes lowers the number of newly formed oligodendrocytes in the spinal cord without affecting proliferation or cell-cycle exit. This effect is due to increased BMP signaling and subsequent increased expression of the oligodendrocyte maturation inhibiting promoter ID2 (Ornelas et al., 2020; Figure 2). These data on the effect of reduced mTOR signaling, in conjunction with other recent evidence, suggest that mTOR signaling must be finely controlled during oligodendrogenesis as increasing mTOR signaling also led to abnormal oligodendrogenesis in mice. Tuberous sclerosis complex-1 (TSC1) reduction results in hyperactive mTOR signaling and oligodendrocyte cultures from Tsc1-cKO mice have significantly lower expression of myelin genes *in vitro*, indicating that increased mTOR signaling may be influencing preOL survival or the integration of new oligodendrocytes (Jiang et al., 2016). In Tsc1-cKO mice, OPC number was significantly reduced once OPCs began active differentiation in the perinatal stages, indicating that cells may be lost in the preOL stage shortly after differentiation. In support of this hypothesis, the increased cell death in *Tsc1* mutants is largely due to apoptosis in the preOL stage as differences in cell death could only be detected in *in vitro* cultures undergoing differentiation. Using RNA-sequencing, the authors found that a cellular stress sensor (CHOP), Fas, and members of the proapoptotic Bcl-2 family were upregulated in Tsc1-cKO mice whereas genes involved in myelination and lipid synthesis were downregulated. These data suggest that hyperactive mTOR signaling increases ER stress and proapoptotic signaling in preOLs and may reduce the ability of preOLs and new oligodendrocytes to engage in lipid synthesis and integrate as mature myelinating oligodendrocytes. Furthermore, ER stress and proapoptotic signaling appear to be dynamically controlled in preOLs by the opposing actions PERK/eIF2α and mTOR signaling. A proper balance between these opposing signaling pathways seems necessary to ensure survival of preOLs (Jiang et al., 2016; Figure 2).

Another intrinsic signaling pathway that modulates preOL survival and integration is mitochondrial stress. Glutathione S-transferase 4α (GSTA4) is an enzyme that regulates oxidative stress by reducing levels of 4-hydroxynonenal (4-HNE) (Bruns et al., 1999). Interestingly, *Gsta4* expression steadily increases as OPCs differentiate into preOLs and subsequently mature into myelin forming oligodendrocytes (Marques et al., 2016; Carlstrom et al., 2020). Oligodendrocytes from *Gsta4* overexpressing rats (DAGsta4) were cultured and it was determined that *Gsta4* overexpression results in more *Plp1* mRNA and fewer OPCs. These data suggest that there is an increase in oligodendrogenesis with no increase in OPC proliferation, resulting in a depleted pool of OPCs. Using RNA-sequencing, the authors show DAGsta4 animals have lower levels of *Fas*, Caspase-8 (*Casp8*) and 4-HNE suggesting that *Gsta4* overexpression lowers mitochondrial 4-HNE load and downregulates the Fas-Casp8-Bid signaling axis (Carlstrom et al., 2020; Figure 2). GSTA4 acts to increase oligodendrogenesis by reducing mitochondrial stress and pro-apoptotic signaling in differentiating oligodendrocytes.

Taken together, these studies highlight the role of intrinsic apoptotic signaling in premyelinating oligodendrocytes and provide evidence that apoptosis of preOLs can influence the rate of oligodendrogenesis. Generally, there are two potential explanations for altered rates of oligodendrogenesis, the first being an increased rate of OPC differentiation, the second an increased rate of preOL survival and integration. Insight can be gained by analyzing both the differentiation rate of OPCs and the survival rate of preOLs in similar studies in the future. Furthermore, measuring the rate of both processes can increase our understanding of the factors that influence oligodendrogenesis in both health and disease.

### Metabolic Demands of Premyelinating Oligodendrocytes

As OPCs differentiate into preOLs, they undergo profound changes in morphology over several days (Figure 1). If preOLs pass the survival checkpoint, they undergo a dramatic increase in total membrane surface area as the cells begin to form myelin sheaths. These dramatic morphological changes require a high energy demand and metabolic stress and profound changes in the epigenetic landscape of the cell (Emery and Lu, 2015). As preOLs transition to new oligodendrocytes, they engage myelin synthesis pathways, and a proper supply of oxygen and metabolites becomes necessary for survival. In fact, evidence indicates that preOLs are considerably more vulnerable to hypoxia-ischemia than OPCs or mature OLs (Cai et al., 2018). In addition, the stabilization of OPC-encoded hypoxia-inducible factor (HIF), whose function is necessary for angiogenesis and myelination in the corpus callosum, in OPCs resulted in maturation arrest, suggesting HIF may play a role in the preOL survival and maturation (Yuen et al., 2014; Figure 2). These data suggest that a regular supply of oxygen is necessary for preOL survival and maturation. It is also becoming evident that the constant supply of key metabolites is required during preOL maturation. Levels of taurine, an important metabolite in the lipid synthesis pathway, have been found to significantly increase as oligodendrocytes differentiate and mature (Beyer et al., 2018). The addition of exogenous taurine to oligodendrocyte cultures enhanced the maturation and survival of OPCs and preOLs, with the most profound effect occurring when taurine was added during the preOL stage (Beyer et al., 2018; Figure 2). Another metabolite, folate, was found to promote OL maturation and survival both *in vivo* and *in vitro* by activating AMPKα phosphorylation (Weng et al., 2017; Figure 2). These studies illustrate the importance of a proper supply of oxygen and myelin building blocks for the survival and integration of preOLs.

The vulnerability of preOLs to low oxygen and metabolites might lead to the hypothesis that fasting or caloric restriction would result in reduced oligodendrogenesis, however, this does not appear to always be true. Reducing the availability of metabolites via fasting has been shown to affect oligodendrogenesis in specific contexts. Alternate day fasting for 6 months increased the maturation of OPCs into MBP + oligodendrocytes (Neumann et al., 2019). Counterintuitively, OPCs from fasting animals have lower expression of differentiation genes and higher ATP levels. One might expect lower levels of differentiation genes in OPCs to result in fewer mature oligodendrocytes, however, there may be a more efficient production of MBP + oligodendrocytes indicating that fasting, and increased ATP levels, may be influencing the survival and maturation of preOLs as opposed to increasing differentiation. This study also shows that metformin can mimic the effects of fasting by increasing levels of phosphorylated AMPK, an effect similar to that of the metabolite folate (Neumann et al., 2019; Figure 2). Interestingly, different durations of dietary restriction seem to have different effects on oligodendrogenesis. Our group found that short term caloric restriction has no effect on oligodendrogenesis (Bacmeister et al., 2020). These results highlight that the effect of diet on oligodendrogenesis is not straightforward and additional studies will likely provide insights into the role of dietary restriction on preOL survival and maturation.

## Extrinsic Mechanisms Regulating Premyelinating Oligodendrocyte Survival and Integration

While intrinsic programmed cell death can exert powerful control over cell fate, there is mounting evidence suggesting that sensory experience, learning, and the local cellular environment can also modulate cell survival. During the last two and a half decades, evidence has emerged suggesting that a wide variety of extrinsic factors such as neuronal activity, neurotransmitters, other glial cells, growth factors, learning, and sensory experience can influence oligodendrocyte biology. While our understanding of how these factors influence oligodendrogenesis at large is understood to some extent, investigations into how these factors affect the survival and integration of preOLs are relatively lacking.

### Neuronal Activity Influences Oligodendrogenesis

**FIGURE 3:**
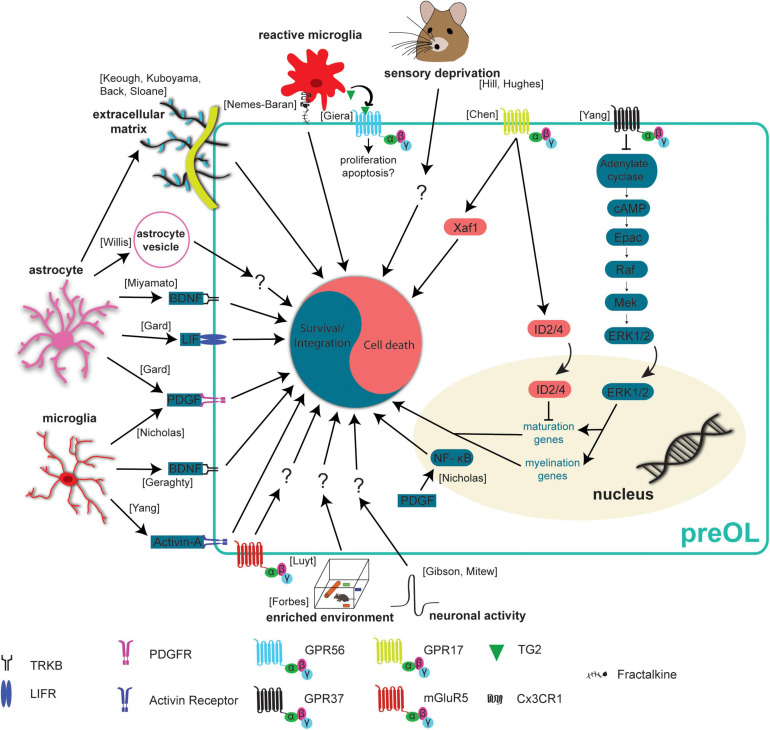
Extrinsic mechanisms regulating preOL survival and integration. Premyelinating oligodendrocytes are responsive to extrinsic signals in their local environment. This means that neurons, microglia, and astrocytes all influence preOLs, in some cases to promote survival and integration (blue) and in others to promote cell death (red). Neuronal activity has been found to increase the production of new oligodendrocytes (Gibson et al., 2014; Mitew et al., 2018). Exposure to enriched environments promotes the production and maturation of new oligodendrocytes after hypoxic injury (Forbes et al., 2020). Sensory deprivation via whisker trimming reduces the production of new oligodendrocytes (Hill et al., 2014; Hughes et al., 2018). Microglial derived factors like Activin-A, PDGF, and BDNF promote survival and integration (Nicholas et al., 2001; Miron et al., 2013; Geraghty et al., 2019). Astrocyte derived factors BDNF, LIF, and PDGF also promote survival and integration (Gard et al., 1995; Miyamoto et al., 2015). PDGF upregulates NF- κB to reduce apoptosis and enhance maturation (Nicholas et al., 2001). The presence of extracellular vesicles from young astrocytes results in more efficient differentiation and maturation of primary OPC cultures (Willis et al., 2020). Activation of mGluR5 receptors reduces staurosporine induced apoptosis (Luyt et al., 2006). Dense extracellular matrix has a negative effect on the production of new oligodendrocytes (Back et al., 2005; Sloane et al., 2010; Keough et al., 2016; Kuboyama et al., 2017). GPR37 signaling results in increased expression of maturation and myelination promoting genes (Yang et al., 2016). Signaling through GPR17 leads to increased nuclear ID2/4 inhibiting the expression of maturation promoting genes (Chen et al., 2009). GPR17 signaling also increases levels of the pro-apoptotic protein, Xaf1 (Chen et al., 2009). Reactive microglia can recognize and engulf preOLs, an effect mediated by the Cx3CR1 receptor and its ligand fractalkine (Nemes-Baran et al., 2020). Reactive microglia also release TG2 which activates GPR56 and potentially reduces the susceptibility of preOLs to apoptosis (Giera et al., 2018).

Neuronal activity exerts great control over an abundance of processes in the CNS from development into adulthood. It is essential for circuit development, neurogenesis, migration, synaptic plasticity, and there is emerging evidence of its role in oligodendrogenesis. Furthermore, while neuronal activity can increase the differentiation of OPCs, it remains less clear whether neuronal activity can modulate the rate at which preOLs survive and integrate into neuronal circuits. One reason for this is that the modes of communication between premyelinating oligodendrocytes and neurons remains undefined. One form of communication between neurons and oligodendrocyte precursors is direct synapses. OPCs form bona fide synapses with neurons, allowing for direct OPC-neuronal communication (Bergles et al., 2000; Lin and Bergles, 2004). However, these synapses are rapidly lost as OPCs differentiate into preOLs, indicating that neuronal activity may influence preOL survival and integration through additional mechanisms, such as extra synaptic communication or growth factors (Kukley et al., 2010; De Biase and Bergles, 2011). Sensory deprivation via whisker trimming reduced the density of mature oligodendrocytes in the somatosensory cortex of early postnatal mice. Genetic fate mapping and *post hoc* immunostaining in slice cultures suggested whisker trimming led to decreased integration of oligodendrocytes (Hill et al., 2014; Figure 3). On the other hand, increased sensory experience via environmental enrichment was found to promote the maturation and generation of new oligodendrocytes during development and after hypoxic injury (Forbes et al., 2020; Goldstein et al., 2021; Figure 3). Furthermore, increasing neuronal activity via optogenetic stimulation increases OPC differentiation and increases the number of newly formed oligodendrocytes (Gibson et al., 2014; Figure 3). Our group showed that motor learning both increases and decreases the rate of oligodendrogenesis in the primary motor cortex depending on the phase of motor training. Specifically, the rate of OPC differentiation and oligodendrogenesis increased in the weeks after motor learning (Bacmeister et al., 2020). However, we found a transient learning-induced suppression of oligodendrogenesis and unaffected OPC differentiation during motor training. These findings suggest that learning may temporarily modulate the survival and integration of preOLs. On a single axon level, increasing neuronal activity of a subpopulation of callosal axons with DREADDs yields increased oligodendrogenesis and increased myelination in an axon selective manner (Mitew et al., 2018; Figure 3). In a similar study, DREADDs were used to specifically stimulate a subpopulation of PV-INs and stimulated neurons had increased axonal growth and increased myelination compared to unstimulated neurons, however, the overall amount of myelination stayed the same (Stedehouder et al., 2018). While it is unequivocal that neuronal activity influences oligodendrogenesis, additional insights into how neurons regulate preOL survival, integration, gene expression, and axonal targeting will greatly increase our understanding of this transient stage of oligodendrogenesis and its role in brain function.

### Premyelinating Oligodendrocyte-Axon Interactions

In the late 1990s, it was proposed that preOLs that were contacting neighboring axons went on to survive, whereas those without processes contacting axons exhibited signs of apoptosis and degeneration (Trapp et al., 1997). This led to the hypothesis that preOL-axon interactions are acting to influence the integration of preOLs into mature oligodendrocytes by modulating the formation and stabilization of nascent myelin sheaths. Recently, zebrafish have proved to be a powerful model for studying the dynamics of nascent sheath formation on axon subtypes. Reducing neuronal activity with tetrodotoxin reduces the proportion of nascent sheaths wrapping axons in the developing zebrafish spinal cord and blocking vesicle release with Tetanus Toxin in single axons reduced myelin wrapping on those axons (Hines et al., 2015; Mensch et al., 2015; Figure 3). A recent study provides further evidence that the release of vesicles from axons promotes the formation and elongation of nascent sheaths (Almeida et al., 2020). These data suggest that vesicle release from axons can modulate nascent sheath dynamics. In fact, the post-synaptic scaffold protein PSD-95 has been observed in myelin forming oligodendrocytes both at the end of myelin sheaths and along their length (Hughes and Appel, 2019; Figure 3). Additionally, there is evidence that high frequency Ca^2+^ transient activity in myelin sheaths results in faster elongation of sheaths, implicating Ca^2+^ signaling in the process of nascent sheath stabilization (Baraban et al., 2018). All these data together support the hypothesis that axons communicate with nascent myelin sheaths and influence their stability and growth. Whether or not this communication influences preOL survival and integration is unknown. Furthermore, investigating what types of axon-preOL communication are necessary for preOL survival could provide insight into the mechanisms governing preOL nascent sheath formation.

Individual oligodendrocytes myelinate multiple axons in the neocortex and the placement of myelin appears to be tightly regulated in healthy animals. These data have led to the hypothesis that neuron-oligodendrocyte communication can both attract and repel the formation of new nascent myelin sheaths. One explanation for the fine network of radial processes observed on preOLs is that they may be surveying the local area for specific neuronal signals to initiate the integration process (Figure 1). Some of these attractive and repulsive cues have been identified in recent years. Neuronal junction adhesion molecule 2 (JAM2) has been identified as an inhibitory molecule that can prevent myelination of neuronal cell bodies (Redmond et al., 2016) and the absence of Neurofascin in oligodendrocytes in zebrafish leads to mistargeting of myelin to cell bodies (Klingseisen et al., 2019). Another study disrupted the paranodal axo-glial junctions using *cntn1b–/–mag–/–* and *caspr–/–mag–/–* mutant zebrafish resulting in hypomyelination and myelin mistargeting (Djannatian et al., 2019). Additionally, normal function of another cell adhesion molecule, Cadm4, is necessary for proper myelination in mice (Elazar et al., 2019). Overall, the identification of attractive and repulsive cues that modulate nascent sheath dynamics is an exciting area of study. Future work linking the molecular mechanisms of axonal myelination to preOL survival and integration will provide a more holistic view of how axon interactions regulate oligodendrogenesis and myelination.

### Glia-Oligodendrocyte Interactions

Just as neuronal activity influences oligodendrogenesis, cell-cell interactions with other glial cell types also regulate myelination. Microglia are the resident immune cells of the CNS and are known to phagocytose synapses and entire cells during periods of plasticity. There is emerging evidence that microglia also influence oligodendrogenesis. However, the role of microglia in oligodendrogenesis is not unidirectional and depends on microglial activation, age, and brain region. The presence of a transient, reactive, ameboid population of microglia in white matter regions during the peak of postnatal myelination led to the hypothesis that microglia may be playing an active role in early postnatal myelin formation. To test this hypothesis, microglia were ablated by application of BLZ945, a CSF-1R inhibitor, from P2 to P7. Microglial ablation resulted in a reduced number of mature oligodendrocytes in the cerebellum and corpus callosum without affecting OPC, astrocyte, or neuronal cell number (Hagemeyer et al., 2017). On the contrary, when 6–8-week-old mice were treated with BLZ945 the opposite effect was seen with a reduction in OPC number and no change in the quantity of mature oligodendrocytes. It is clear that microglia influence the oligodendrocyte lineage in both young and adult animals and future work can provide deeper insight into the effect of microglial ablation on the preOL stage of oligodendrogenesis. While microglial ablation can provide insights into the larger role of microglia in oligodendrogenesis, investigations of microglia-oligodendrocyte interactions can elucidate the role of microglia on the level of single oligodendrocytes and even single myelin sheaths.

To study the microglia-oligodendrocyte interaction, *ex vivo* brain slices and confocal imaging were used to track microglia engulfment of oligodendrocytes in the corpus callosum. Microglia appear to engulf viable OPCs and preOLs from P4-P11, an effect that is reduced when CX3C chemokine receptor 1 (CX3CR1) is conditionally knocked out of microglia (Nemes-Baran et al., 2020; Figure 3). These results indicate that there may be higher rates of preOL survival and integration when CX3CR1 is knocked out in microglia, but further work is necessary to fully understand the interactions between microglia and preOLs. Other recent work has utilized electron microscopy and live imaging to investigate the interactions of microglia with single oligodendrocytes and sheaths. Djannatian and colleagues used electron microscopy of the P14 optic nerve to provide evidence that microglia phagocytose abnormal myelin during development in mice and there is accumulating evidence that microglia survey myelin sheaths and phagocytose myelin in the zebrafish spinal cord during development (Hughes and Appel, 2020; Djannatian et al., 2021). Taken together, these data show that microglia interact with myelin sheaths during development and help to ensure proper myelination.

Evidence is accumulating that microglia can influence oligodendrogenesis and preOLs through the release of a wide range of factors into the local microenvironment. Homeostatic microglia have been shown to promote the survival and maturation of OPCs and potentially preOLs *in vitro*. Oligodendrocyte cultures supplemented with media from homeostatic microglial cultures had reduced apoptosis compared to cultures with control media, an effect that is blocked by removing Platelet derived growth factor (PDGF) from the media (Figure 3). Furthermore, PDGF may promote the survival of cultures oligodendrocytes by upregulating Nuclear Factor κB (NF- κB), as activating NF- κB in cultures was sufficient to reduce apoptosis and enhance maturation (Nicholas et al., 2001). Homeostatic control of OPC density through proliferation is necessary to ensure that OPCs are available to form new oligodendrocytes throughout the CNS (Hughes et al., 2013). A recent study used microglial specific deletion of neuropilin-1 (Nrp1) to show that microglial derived Nrp1 is necessary for OPC proliferation, expansion and subsequent oligodendrocyte formation (Sherafat et al., 2021). Another microglial derived factor, Activin-A, was recently found to influence oligodendrogenesis *in vitro*. Activin-A containing media from microglia promotes the production of MBP + oligodendrocytes *in vitro* (Miron et al., 2013; Figure 3), suggesting that Activin-A signaling through activin receptors leads to increased differentiation of OPCs, increased survival and integration of preOLs, or both. Another population of microglia expressing CD11c^+^ were recently found to be the primary source of insulin-like growth factor 1 (IGF1) and deletion of *Igf1* from this population resulted in significant myelin deficiencies (Wlodarczyk et al., 2017). While microglial derived factors that benefit oligodendrocyte maturation, survival and integration have been found *in vitro*, whether these factors have the same effect *in vivo* is less clear. In addition to testing the role of these factors in oligodendrogenesis *in vivo*, it would be beneficial to investigate the role of PDGF, NF- κB, and Activin-A at the different stages of oligodendrogenesis by adding it to cultures when cells are in the OPC, preOL, or newly formed oligodendrocyte stages, respectively.

Astrocytes tile the CNS allowing them to survey and modulate extracellular microenvironments and release factors to influence cells in close proximity. Interestingly, oligodendrocytes and astrocytes are directly coupled by gap junctions, forming a glial syncytium allowing for fast communication and transfer of molecules between these cell types (Orthmann-Murphy et al., 2008). The connexins Cx47 and Cx32 are expressed by oligodendrocytes and mediate the formation of specific astrocyte-oligodendrocyte gap junctions. Recent RNA-sequencing studies provided evidence that the expression of these connexins begins as preOLs integrate as new oligodendrocytes, raising the possibility that these gap junction connections may be necessary for the survival and integration of preOLs (Zhang et al., 2014; Figure 1). Beyond physical connections, astrocytes can also provide additional factors to influence oligodendrocytes. Astrocyte-derived BDNF supports oligodendrogenesis after white matter injury (Miyamoto et al., 2015; Figure 3). Furthermore, genetic elimination of the TrkB receptor from OPCs prevents activity dependent OPC proliferation and adaptive myelination in response to increased neuronal activity (Geraghty et al., 2019). However, whether BDNF promotes the survival of preOLs or just increases OPC differentiation remains to be addressed. Two other growth factors that can be released by astrocytes, PDGF and Leukemia inhibitory factor-like protein, also regulate oligodendrogenesis. In cell culture medium conditioned by astrocytes, differentiated oligodendrocytes survived for weeks whereas oligodendrocytes cultured in non-conditioned media died 1–2 days after differentiation, and the increased survival is likely due to the action of PDGF and/or leukemia inhibitory factor as removal of these factors reduced survival of differentiating oligodendrocytes (Gard et al., 1995; Figure 3). In addition to the release of growth factors, astrocytes can influence oligodendrocytes through the release of vesicles. Primary OPCs cultured in the presence of extracellular vesicles from young astrocytes more efficiently differentiated and matured into myelin forming oligodendrocytes compared to OPCs cultured in control media or media with vesicles from aged astrocytes (Willis et al., 2020; Figure 3). This suggests that astrocytic vesicles contain some type of signaling molecules that may influence preOL survival and integration. Future work regulating or blocking release of astrocyte vesicles *in vivo* and investigating the effects on oligodendrogenesis would be insightful. Together, these studies indicated that astrocyte-preOL interactions may play a pivotal role in survival and integration of preOLs during oligodendrogenesis.

### G Protein-Coupled Receptors (GPCRs)

PreOLs express a wide range of receptors on the cell surface and the expression of these receptors changes rapidly as differentiation progresses and the cells mature into myelin forming oligodendrocytes. One subgroup of receptors, the G protein-coupled receptors, have been shown to influence multiple aspects of oligodendrogenesis. Three GPCRs, GPR17, GPR56, and GPR37 all exhibit peak expression at different stages of oligodendrogenesis, potentially exerting control on the process at specific stages of maturation. GPR17 rapidly increases in expression as OPCs begin the differentiation process and expression stays high as these cells integrate and mature as myelinating oligodendrocytes (Marques et al., 2016). GPR17 negatively regulates oligodendrocyte maturation and inhibits survival through a few distinct mechanisms. Genetic deletion of GPR17 in knockout mice exhibit accelerated maturation of oligodendrocytes which can be attributed to increased expression and nuclear translocation of ID2/4 (Chen et al., 2009; Figure 3). On the other hand, sustained activation of GPR17 resulted in impaired oligodendrocyte survival via increased expression of the proapoptic factor *Xaf1* and reduced intracellular cAMP levels (Ou et al., 2016). GPR56 is expressed in OPCs and preOLs but expression slowly rises and peaks in new oligodendrocytes (Marques et al., 2016). Microglia can influence preOL survival and integration through microglial derived Tranglutaminase-2 (TG2) which activates GPR56 on OPCs. TG2/laminin/GPR56 signaling increases OPC proliferation and improves remyelination in two different mouse models of demyelination (Giera et al., 2018; Figure 3). This is especially interesting because TG2-laminin-GPR56 signaling has been shown to inhibit apoptosis in other cell types (Olaniru et al., 2018) making it plausible that GPR56 may inhibit apoptosis in preOLs. GPR37 expression is low in OPCs and preOLs but increases very rapidly as preOLs integrate and mature into newly formed oligodendrocytes (Marques et al., 2016). A recent report highlights GPR37 as a negative regulator of oligodendrocyte maturation. Genetic deletion of GPR37 increases the maturation of preOLs and results in hypermyelination. These effects are mediated through the ERK1/2 signaling pathway (Yang et al., 2016; Figure 3).

Another type of GPCR, the metabotropic glutamate receptor, has been shown to modulate oligodendrocyte survival. Metabotropic glutamate receptor 5 (mGluR5) is downregulated in preOLs and its expression peaks in new oligodendrocytes suggesting it may play a role in the survival of preOLs. In fact, activation of mGluR5 substantially reduced staurosporine-induced apoptosis of oligodendrocytes cultured *in vitro*, suggesting that reduced expression of mGluR5 in preOLs may contribute to their vulnerability to apoptosis (Luyt et al., 2006; Figure 3). While it is clear that signaling through GPCRs can influence oligodendrogenesis, many of these studies activate or inhibit GPCR signaling in the entire oligodendrocyte lineage rather than in preOLs specifically. Developing tools that are specific to preOLs would allow for the investigation of the role of these receptors with cell stage specificity and help to tease apart how receptor signaling influences preOL survival and integration.

### Extracellular Matrix

The extracellular matrix (ECM) exerts control over many processes in the CNS including structural and functional plasticity of neurons (Lauri et al., 1998; Dityatev et al., 2010; Kochlamazashvili et al., 2010) as well as myelination (Harlow and Macklin, 2014). ECM is produced by many different cells in the CNS including neurons, astrocytes, and even OPCs, which are known to produce some types of ECM such as Neural/glial antigen 2 (NG2) and laminin (Yang et al., 2006; Lam et al., 2019). Generally, deposition of ECM components into a local environment acts to limit the extension of cellular processes, which could be detrimental to the rapid changes in morphology observed during preOL survival and integration. In fact, chondroitin sulfate proteoglycan (CSPGs) reduce the growth of OPCs in culture and treatment with Fluorosamine, a compound that inhibits astrocyte synthesis of CSPGs, increases the number of mature oligodendrocytes in a demyelinated lesion without affecting the number of OPCs (Keough et al., 2016). This indicates that preOLs may survive and integrate at higher rates in the absence of dense ECM. In support of this hypothesis, culturing OPCs on dishes coated with increasing levels of aggrecan revealed that aggrecan reduces the ratio of MBP + /NG2 + cells, indicating a reduction in oligodendrogenesis. Addition of protamine, a ligand for the protein tyrosine phosphatase receptor type Z (PTPRZ), effectively rescued the maturation of oligodendrocytes on aggrecan coated coverslips *in vitro*, however, it is possible that this effect is due to increased differentiation of OPCs or increased preOL survival and integration (Kuboyama et al., 2017). It may be beneficial and insightful to investigate if reducing CSPGs *in vivo* increases preOL survival and integration by making the extracellular environment more permissive to preOLs process extension, perhaps by observing the ability of preOLs to extend processes in high and low CSPG environments. Another ECM component produced by astrocytes, hyaluronan, was found to inhibit oligodendrocyte maturation *in vitro.* Removing hyaluronan from cultures increased the percentage of O4 + oligodendrocytes, indicating increased maturation of differentiated OPCs into preOLs (Back et al., 2005). The repressive effects of hyaluronan on oligodendrocyte maturation was later found to act through Toll-like receptor 2 (TLR2) and MyD88 signaling in oligodendrocytes (Sloane et al., 2010). These studies clearly show that the ECM plays an important role in oligodendrogenesis by inhibiting the differentiation of OPCs and perhaps also influencing the survival and integration of preOLs (Figure 3).

## Premyelinating Oligodendrocytes in Multiple Sclerosis and Drug Development

Multiple sclerosis (MS) is the most common demyelinating disease of the central nervous system in adults and is characterized by a loss of oligodendrocytes and myelin in lesions. Up until recently, treatments for MS have focused on preventing recurrence of the disease by modulating the immune system. Recently, new approaches to increase remyelination in lesions have begun to be explored and tested. While remyelination is absent or limited in the majority of MS lesions (Prineas and Connell, 1979; Yeung et al., 2019), new “remyelination therapies” for MS seek to increase myelin repair in lesions by commandeering and enhancing natural repair mechanisms, especially oligodendrogenesis. The reason for the failure of to repair MS lesions is unknown. Several factors may contribute to this deficiency: inefficient (1) OPC recruitment, (2) OPC differentiation into new oligodendrocytes, or (3) integration of premyelinating oligodendrocytes. Furthermore, there is evidence that OPCs lose their capacity for differentiation with age (Neumann et al., 2019).

The regulation of premyelinating oligodendrocytes within demyelinating lesions of MS patients is not well characterized. Within lesions of some MS patients, the number of preOLs was much higher than healthy conditions, however, these numbers are reduced with disease course progression (Chang et al., 2002). Recent work shows that an intermediate population of oligodendrocytes, potentially premyelinating oligodendrocytes, are greatly reduced in number compared to other types of oligodendrocytes in MS lesions (Jakel et al., 2019). Taken together, these studies of human MS lesions provide evidence that preOLs are present in lesions, albeit reduced in number. However, the capacity of preOLs to survive and integrate in lesions remains unknown. Generally, lesions appear to be relatively inhospitable to the production of new oligodendrocytes which may be attributable to neurodegeneration, inflammation, or a combination of both (Correale et al., 2017). As discussed above, preOLs are especially vulnerable to hypoxia, limited metabolites, active microglia, and neuronal activity, all of which are disrupted in neurodegenerative and inflammatory conditions in MS lesions. It is possible that the disruption of all of these processes makes lesions especially hostile to preOLs and may result in an even greater loss of oligodendrocytes at the preOL stage due to cell death than that observed in healthy animals. Furthermore, there is considerable evidence of pathological differences between white matter lesions and gray matter lesions suggesting that preOL survival and integration may be heterogenous across different brain regions (Prins et al., 2015). The development and translation of new preOL markers into human tissue will allow for more detailed investigations into the biology of premyelinating oligodendrocytes in the context of demyelinating disease.

**FIGURE 4:**
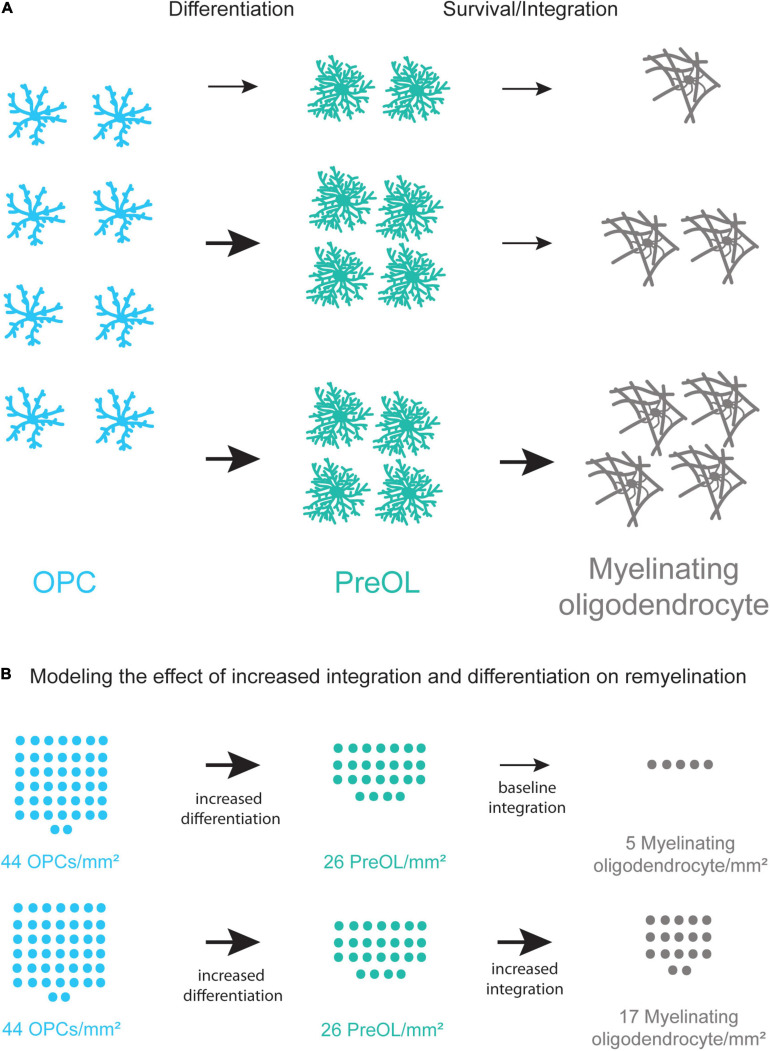
Coupling increased differentiation of OPCs with increased survival and integration of preOLs may lead to more new oligodendrocytes. **(A)** A schematic representation of the remyelination process. Increasing the differentiation rate of OPCs (blue) results in a small increase in new oligodendrocyte formation (gray). Increasing the differentiation rate of OPCs and the survival and integration rate of preOLs (teal) has the potential to increase new oligodendrocyte formation (bottom pathway) more than increasing differentiation alone (middle pathway). **(B)** Modeling the potential increase in new oligodendrocyte formation in chronic MS lesions when coupling increased differentiation with increased preOL survival and integration. Chronic lesions contain around 44 OPCs/mm^2^ (Kuhlmann et al., 2008) yet production of new oligodendrocytes is limited. The modeling represents the potential number of new myelinating oligodendrocytes/mm^2^ at varying rates of differentiation and integration. These rates are pharmacologically-induced differentiation *in vitro* (Mei et al., 2014), and integration of *Bax*–/–*Bak*–/– oligodendrocytes *in vitro* (Kawai et al., 2009). Increasing the rate of preOL survival and integration in conjunction with increased OPC differentiation is predicted to result in substantially more new myelin forming oligodendrocytes in MS lesions.

Stemming from these concepts, many current MS therapies seek to overcome the block of OPC differentiation by modulating OPC differentiation using a pharmacological or behavioral intervention (Deshmukh et al., 2013; Mei et al., 2014; Najm et al., 2015; Bacmeister et al., 2020). Treatments increasing OPC differentiation have had success in animal models and there have been several clinical trials in recent years with promising results, however, there is room for additional improvement (Green et al., 2017; Schwartzbach et al., 2017). Multiple, concurrent approaches to increase oligodendrogenesis in MS patients may be required as recent studies show that the formation of new oligodendrocytes is limited in lesions (Yeung et al., 2019). Furthermore, the lesion environment in different brain regions may require specific approaches as well as the recovery of heterogeneous populations of mature, myelinating oligodendrocytes (Jakel et al., 2019; Floriddia et al., 2020). One potential strategy may be to increase the rate of OPC differentiation while simultaneously promoting preOL survival and integration to increase the efficiency of remyelination (Figure 4). Interestingly, Chang and colleagues observed that preOLs are present in human MS lesions and that they associate with demyelinated axons. However, these preOLs do not myelinate denuded axons, indicating a failure of preOL integration (Chang et al., 2002). While the number of OPCs is reduced in chronic lesions of MS patients, they are still present at around 44 OPCs/mm^2^ indicating there is potential for the differentiation and integration of new myelin forming oligodendrocytes (Kuhlmann et al., 2008). Future studies examining the abilities of current remyelination therapies that promote preOL integration can help increase the extent and efficiency of myelin repair in patients.

Multiple sclerosis is a debilitating and complex disease and recent advances in our understanding of oligodendrocyte cell biology have led to emerging therapies focused on enhancing remyelination in lesions. While these therapies are promising, it is becoming more likely that increasing OPC differentiation on its own may not be enough to sufficiently enhance remyelination in MS patients. Characterizing preOL survival and integration in the different microenvironments of demyelinated lesions in both white matter and gray matter will provide insight into preOL function in demyelination. This knowledge may allow us to develop therapies that make the lesion environment more suitable for preOLs or make preOLs more resistant to the lesion microenvironment. Coupling current therapies that increase OPC differentiation with increased preOL survival and integration has the potential to greatly enhance remyelination.

## Conclusion

Premyelinating oligodendrocytes are the crux of oligodendrocyte differentiation and oligodendrogenesis. Until recently, there have been limited tools to visualize and investigate the mechanisms of preOL survival and integration. While our understanding of OPC and oligodendrocyte biology has advanced rapidly, our knowledge of preOLs remains incomplete. Recent advances in RNA-sequencing, mouse genetics, and advanced imaging techniques have allowed for the visualization and characterization of this elusive stage in oligodendrocyte development. Future development of stage specific tools to visualize and manipulate this transient cellular stage in intact animals will increase our ability to interrogate the cellular mechanisms underlying preOL behavior and may provide insights into how preOLs contribute to learning, memory and cognition. Furthermore, modulation of preOL survival and integration holds great potential to modulate myelination in the context of plasticity in the healthy brain and regeneration following demyelinating injuries and disease.

## Author Contributions

MS and EH conceived the topic, prepared the figures, and wrote the manuscript. Both authors contributed to the article and approved the submitted version.

## Conflict of Interest

The authors declare that the research was conducted in the absence of any commercial or financial relationships that could be construed as a potential conflict of interest.
